# *In vitro* Modeling of Embryonal Tumors

**DOI:** 10.3389/fcell.2021.640633

**Published:** 2021-02-26

**Authors:** Lars Custers, Irene Paassen, Jarno Drost

**Affiliations:** ^1^Princess Máxima Center for Pediatric Oncology, Utrecht, Netherlands; ^2^Oncode Institute, Utrecht, Netherlands

**Keywords:** pediatric cancer, embryonal tumors, *in vitro* models, organoids, therapy

## Abstract

A subset of pediatric tumors affects very young children and are thought to arise during fetal life. A common theme is that these embryonal tumors hijack developmental programs, causing a block in differentiation and, as a consequence, unrestricted proliferation. Embryonal tumors, therefore typically maintain an embryonic gene signature not found in their differentiated progeny. Still, the processes underpinning malignant transformation remain largely unknown, which is hampering therapeutic innovation. To gain more insight into these processes, *in vitro* and *in vivo* research models are indispensable. However, embryonic development is an extremely dynamic process with continuously changing cellular identities, making it challenging to define cells-of-origin. This is crucial for the development of representative models, as targeting the wrong cell or targeting a cell within an incorrect developmental time window can result in completely different phenotypes. Recent innovations in *in vitro* cell models may provide more versatile platforms to study embryonal tumors in a scalable manner. In this review, we outline different *in vitro* models that can be explored to study embryonal tumorigenesis and for therapy development.

## Introduction

Cancer is the leading disease-related cause of death in children (Siegel et al., 2016; Cunningham et al., 2018). A significant subset of pediatric tumors occurs in early childhood, suggestive of an origin in prenatal life (Marshall et al., 2014). These so-called embryonal tumors are thought to develop as a consequence of aberrant development. However, for many embryonal tumors the processes driving tumorigenesis remain unknown. Whereas, adult cancers develop by a progressive accumulation of mutations over many years (Stratton et al., 2009), embryonal tumors are typically characterized by a relatively low mutational burden and only a few genetic events to drive tumorigenesis (Vogelstein et al., 2013; Gröbner et al., 2018; Rahal et al., 2018; Kattner et al., 2019). The few genetic alterations that do occur likely cause fetal cells to maintain a progenitor-like state and prohibit differentiation. This maturation block has been suggested to prime cells for malignant transformation (Chen et al., 2015; Puisieux et al., 2018; Rahal et al., 2018; Jessa et al., 2019). To better understand the processes underpinning embryonal tumorigenesis, a direct comparison between normal and tumor development is key. Gene expression profiling of fetal tissues with single cell resolution has provided more insights into the developmental trajectories driving embryogenesis. Comparison of such profiles with tumor gene expression signatures have defined the cellular identity of several embryonal tumors, possibly pointing to their cellular origin (Boeva et al., 2017; Young et al., 2018, 2020; Hovestadt et al., 2019; Jessa et al., 2019; Vladoiu et al., 2019). Yet, in many cases these studies are merely correlative and lack subsequent functional validation. To do so, representative *in vitro* and *in vivo* preclinical models are crucial.

Genetically engineered mouse models (GEMMs) have been the golden standard for finding the cellular origin of cancers, by introducing tumor driver events in putative tumor-initiating cells (Visvader, 2011; Marshall et al., 2014). Although GEMMs have provided important insights into tumorigenesis, several drawbacks limit their potential as a representative model of embryonal tumors. Embryonic development is an extremely dynamic process with continuously changing cellular identities, which makes it very challenging to target the right cell at the right time. For instance, homozygous loss of the Wilms tumor driver gene *Wt1* was shown to be embryonically lethal in mice (Kreidberg et al., 1993), whereas a specific *Wt1* ablation at E11.5 in a small fraction of nephron progenitor cells resulted in Wilms tumor formation (Hu et al., 2011; Berry et al., 2015; Huang et al., 2016). Moreover, GEMM generation is time consuming and mouse development does not fully recapitulate human embryogenesis (Navin et al., 2010, 2011; Blakeley et al., 2015; Theunissen and Jaenisch, 2017). The development of new *in vitro* cell models increasingly recapitulating the complexity of organogenesis will open new avenues for the development of novel, relevant embryonal tumor models. In this review, we discuss the currently available *in vitro* models to study embryonal tumorigenesis as well as the discovery of new therapeutic strategies.

## Cell Lines of Fetal Origin

A broad range of cell lines has been established over the last decades. Cell lines are easy to maintain and typically do not consume many resources, which allows for fast and parallel modeling of multiple tumor driver events. This is particularly useful to interrogate the complex genetics underlying heterogeneous tumor phenotypes. One such tumor is neuroblastoma, which is characterized by a variety of driver events, including *MYCN* amplification and *ALK* mutations (Johnsen et al., 2019). To study neuroblastoma initiation, models of its embryonic origin, neural crest (Johnsen et al., 2019), are required. *In vitro* murine neural crest models can be generated by extraction of neural tubes from mouse embryos, which are subsequently placed in a culture dish to initiate the migration of neural crest cells onto the plate (Maurer et al., 2007; Olsen et al., 2017). The neural crest cells lose their multipotency over time *in vitro* (6–10 cell divisions) (Stemple and Anderson, 1992) and are, therefore only suitable for short-term experiments. However, multipotency can be maintained by exogenous c-Myc expression. Accordingly, Maurer et al. (2007) generated the JoMa1 neural crest cell line, which was established from mouse embryos carrying the inducible c-MycER transgene, enabling tamoxifen-inducible c-Myc expression and maintenance of multipotency. In both the JoMa1 cell line (Schulte et al., 2013) and non-genetically modified neural crest cells (Olsen et al., 2017), overexpression of *MycN* was proven sufficient to generate neuroblastoma upon transplantation in immune-deficient mice. Other murine neural crest-derived neuroblastoma models accommodate oncogenic variants of *Alk* or *Phox2b*, which was shown to impair neural crest development and inhibit sympathoadrenal differentiation processes (Reiff et al., 2010; Schulte et al., 2013; Montavon et al., 2014). However, murine neural crest development has been shown to be different from human in many aspects (O’Rahilly and Müller, 2007; Betters et al., 2010). Cohen et al. (2020), therefore developed a mouse-human chimera to study neuroblastoma formation in a human setting. Human iPSC-derived neural crest cells were injected *in utero* into gastrulating mouse embryos to form a human neural crest lineage in mice. For neuroblastoma modeling, the neural crest cells were subsequently genetically engineered with inducible expression constructs of *MYCN* and an oncogenic variant of *ALK*. Upon induction, mice developed tumors characteristic of patient neuroblastoma, and tumor transcriptomes resembled neuroblastoma patients more closely than GEMMs. Interestingly, injections subcutaneously lead to tumor formation but without expression of neuroblastoma markers (Cohen et al., 2020). These findings suggest that human neural crest cells serve as a more representative model than mouse, but only when generated in the appropriate developmental context and orthotopic environment.

Another embryonal tumor entity where differences between human and mouse models of tumorigenesis were observed is retinoblastoma. The common driver event of retinoblastoma is loss of *RB1* during retinal development (Dimaras et al., 2015). Retinoblastoma modeling using GEMMs has proven challenging, as engineering of *Rb1*-deficient mice resulted in embryonic lethality (Lee et al., 1992; Wikenheiser-Brokamp, 2006) and retina-specific depletion of *Rb1* was required. However, in contrast to human, mouse retinal cells were proven insensitive to *Rb1* depletion and required additional knock-outs of tumor suppressors *p107* or *p130* for retinoblastoma development (Robanus-Maandag et al., 1998; Dannenberg et al., 2004; MacPherson et al., 2004). To generate human models of retinal development, Xu et al. (2014) isolated human fetal retinal cells post-fertilization retaining all retinal precursor cell types (RPCs) at distinct maturation states. Depletion of *RB1* within the different RPCs indicated post-mitotic cone-precursors to be most prone to develop into retinoblastoma, based on its ability to form tumors with expression of retinoblastoma markers upon xenografting in mice (Xu et al., 2014). Furthermore, *RB1* loss in matured retinal cells did not induce retinoblastoma, validating that tumor initiation is restricted to a specific cell within retinal development.

Overall, *in vitro* modeling of retinoblastoma and neuroblastoma in human and mouse fetal cell cultures uncovered that fundamental differences between mice and human development can impede representative modeling of embryonal tumors.

## Pluripotent Stem Cell-Derived Cell Lines

Classical cell lines are typically composed of a single type of progenitor-like cell representing a specific germ layer (i.e., endoderm, ectoderm, mesoderm, neural crest). Culture models still capable of generating the different germ layers give the opportunity to model embryonal tumors of which it is not yet clear from which lineage they arise, or which seem to arise across the boundaries of the different germ layers. Current *in vitro* models capable of recapitulating these different developmental trajectories include pluripotent stem cells (PSCs) such as embryonic stem cells (ESCs) and induced PSCs (iPSCs), which can self-renew and be subjected to differentiation protocols that enforce all germ layers (Liu G. et al., 2020). PSCs can be stably maintained in culture and are permissive for genetic manipulation (Liu G. et al., 2020). With the development of effective differentiation protocols, PSCs can mirror embryonic development and therefore serve as a valuable model to study tumorigenesis. iPSCs are generated through the forced dedifferentiation of somatic cells, which thereby regain pluripotency. The molecular mechanisms that underly this reprogramming show significant similarities with the processes driving a subset of the embryonal germ cell tumors (GCTs) (Oosterhuis and Looijenga, 2019), including yolk sac tumors, embryonal carcinomas, and teratomas. GCTs encompass a diverse group of cancer entities that arise from cells of the early embryo or germ line (Oosterhuis and Looijenga, 2019). Interestingly, somatic mutations play a minor role as drivers of GCT development. Tumors are thought to arise by epigenetic deregulation of the cell-of-origin or aberrant stem cell niche factors (Oosterhuis and Looijenga, 2019). The developmental potency of the cell-of-origin can be reprogrammed through increased expression of well-known pluripotency factors, such as NANOG and OCT4 (De Jong and Looijenga, 2006; Thomas et al., 2011). Xenograft studies have shown that iPSCs and ESCs are intrinsically tumorigenic (Ben-David and Benvenisty, 2011). Upon xenografting, iPSCs develop into a benign GCT referred to as teratoma or in some cases more malignant GCTs, dependent on the reprogramming method applied (Lee et al., 2013). These findings indicate that maintaining an early embryonic cellular context is, by itself, sufficient for tumor initiation. Although PSC tumorigenicity is a limitation for its potential application in regenerative medicine, iPSCs and ESCs can on the other hand serve as *in vitro* models of GCTs.

A major class of genes mutated in childhood as well as adult cancers are subunits of the SWItch/Sucrose Non-Fermentable (SWI/SNF) chromatin remodeling complex (Wilson and Roberts, 2011; Shain and Pollack, 2013). The role of this complex in embryonal tumors is clearly exemplified in malignant rhabdoid tumors (MRT), which are characterized by the complete loss of SWI/SNF subunit *SMARCB1* (95% of cases) or *SMARCA4* (5% of cases) (Lee et al., 2012; Hasselblatt et al., 2014). To study MRT initiation, *SMARCB1* was knocked down in hESCs using RNA interference (Langer et al., 2019). The differentiation capacity of hESCs was subsequently assessed, demonstrating that *SMARCB1* inhibition specifically repressed neural induction, whereas mesodermal and endodermal lineage induction was not affected (Langer et al., 2019). In culture conditions inducing neural differentiation, *SMARCB1* was shown to be essential for increased chromatin accessibility at neural differentiation genes and silencing of pluripotency-related super-enhancers (Wang et al., 2017; Langer et al., 2019). Furthermore, *SMARCB1*-null iPSCs that were transplanted into mice were able to generate MRT (Terada et al., 2019). Interestingly, iPSCs that had further progressed to neural progenitor cells (NPCs) generated tumors without rhabdoid features. These results show a lineage-specific role for *SMARCB1 in vitro*, validating recently developed MRT GEMMs wherein *Smarcb1* loss-induced rhabdoid tumor development was demonstrated to be limited to a specific developmental time and lineage (Han et al., 2016; Vitte et al., 2017).

A different layer of epigenetic regulation affected in embryonal tumors is the post-translational modification of histone tails, which enables a rapid switch between active or repressive histone marks to dynamically regulate gene expression during development. Mutations in histones are specifically characterized in a subset of pediatric gliomas. In diffuse intrinsic pontine glioma (DIPG), nearly 80% of cases have a missense mutation in the histone 3.3 gene (*H3F3A*), causing a substitution of methionine for lysine 27 (H3K27M) (Khuong-Quang et al., 2012; Schwartzentruber et al., 2012; Wu et al., 2012). The origin of DIPG was indicated to lie in early neural development (Filbin et al., 2018; Sun et al., 2019), presumably making NPCs derived from ESCs a suitable model for tumor initiation. In line with this, overexpression of the H3K27M mutant in NPCs resulted in increased proliferation (Funato et al., 2014). Interestingly, introduction of the mutation was ineffective in uninduced ESCs or mature astrocytes. For a majority of DIPG cases, H3K27M mutations are typically co-occurring with amplification of *PDGFRA* and loss of *TP53* (Khuong-Quang et al., 2012). Combined introduction of these three genetic events in NPCs induced more extensive neoplastic features, generating DIPG when transplanted in mice (Funato et al., 2014). This combination of mutations prohibited early NPCs to differentiate to astrocytes (Funato et al., 2014), explaining the observed maturation block in DIPG.

MRT and DIPG modeling approaches using ESCs and iPSCs have demonstrated that a specific cellular context is required for malignant transformation, meaning that tumorigenesis is restricted to a specific developmental time and fetal cell type.

## Pluripotent Stem Cell-Derived Organoids

Recent innovations in three-dimensional (3D) culture technology, such as organoids, has opened new opportunities for generating additional representative models of embryonal tumors. Organoids can be derived from adult (ASC) or pluripotent stem cells. They typically capture the cellular and genetic heterogeneity of native tissue and recapitulate cellular hierarchy and dynamics to a large extent, which is most likely a consequence of their 3D architecture (Clevers, 2016). Therefore, 3D organoid cultures seem to better recapitulate organ morphogenesis (Clevers, 2016).

Following that rationale, 3D retinal organoids were established from hESCs or iPSCs, allowing for more comprehensive studies of retinoblastoma initiation in human cells (Zhong et al., 2014; Kuwahara et al., 2015). Loss of *RB1* in retinal organoids showed a dysregulation of retinal maturation processes, impairing differentiation toward photoreceptors, ganglion, and bipolar cells (Zheng et al., 2020). However, the depletion of *RB1* was not sufficient for retinoblastoma initiation as the organoids did not fully recapitulate the retinoblastoma cell phenotype. In addition, transplantation of *RB1*-null organoids into immune-deficient mice did not result in retinoblastoma formation (Zheng et al., 2020). In contrast, Liu H. et al. (2020) utilized an alternative hESC-derived retinal organoid model, in which *RB1* depletion did successfully generate tumors upon xenografting and better resembled patient retinoblastoma. These findings illustrate that the finetuning of retinal organoid establishment can affect the outcome of *RB1* depletion, possibly due to differences in cellular composition and the presence or absence of the cell-of-origin. These studies further highlight the specific cellular context required for retinoblastoma initiation and point out a possible limitation of PSC-derived models, as they may not be able to generate the full extent of cell-types found *in vivo*.

A frequent source of embryonal tumors is the embryonic brain. Human brain development can be mimicked by differentiation of PSCs to neural progenitor cells. In culture, they can self-organize into cerebral or cerebellar organoids containing different cell types in a polarized structure (Muguruma et al., 2015; Luo et al., 2016). Embryonic cerebellar organoids have been successfully used to model pediatric brain tumors, including medulloblastoma and rhabdoid tumors (Ballabio et al., 2020; Parisian et al., 2020). Organoid cultures can be utilized to introduce tumorigenic mutations in a systematic manner, as shown for cerebral organoids (Bian et al., 2018), demonstrating the potential for high-throughput *in vitro* tumor modeling. Furthermore, cerebellar organoids can be exploited to decipher tumor subtype-specific processes. Medulloblastoma, among other embryonal tumor entities, is classified into subtypes based on the oncogenic activation of specific signaling pathways (Cavalli et al., 2017). The medulloblastoma subgroup 3 (*MYC* amplified subgroup) was successfully modeled in cerebellar organoids by combination of *MYC* and *OTX2* or *GFI1* overexpression (Ballabio et al., 2020). The genetically modified cerebellar organoids showed increased proliferation and enrichment for progenitor cells, indicative of a differentiation block. Upon transplantation into mice, medulloblastomas developed resembling subgroup 3 tumors based on marker genes and DNA methylation patterns. Other medulloblastoma subtypes, likely arising from distinct neural differentiation trajectories, have not been modeled *in vitro* up to date (Gibson et al., 2010; Grammel et al., 2012; Hovestadt et al., 2019). To do so, tumor initiation models composed of different neural lineages may be required.

Overall, the development of embryonic organoid cultures has provided relevant models of embryonal tumorigenesis. By approaching *in vivo* physiology, human organoids may serve as a promising alternative for time- and labor-intensive *in vivo* studies.

## Reverse Tumor Modeling and Differentiation Therapy

Relieving the differentiation block underpinning embryonal tumor development could potentially serve as a therapeutic approach (i.e., maturation therapy). To develop such therapies, the differentiation block must first be defined, which can be achieved through reverse tumor modeling by, for instance, reverting the oncogenic driver in cultured tumor cells. Following this principle, inhibition of N-MYC in *MYCN*-amplified neuroblastoma cell lines induced a differentiation morphology as well as upregulation of neural differentiation genes (Kang et al., 2006; Henriksen et al., 2011; Jiang et al., 2011; Westermark et al., 2011; Hossain et al., 2013). Differentiation phenotypes were also observed upon genetic manipulation of medulloblastoma models (Liu et al., 2017; Cheng et al., 2020; Zagozewski et al., 2020), and MRT models (Betz et al., 2002; Nakayama et al., 2017; Wang et al., 2017). These studies show that reversal of the genetic driver can transform tumor cells to a more mature cell state, possibly reflecting the matured cell type it would have become, had it not become cancerous. Genetic repair of driver genes is not feasible at present (Dunbar et al., 2018). An alternative strategy is to induce differentiation pharmacologically. For instance, experiments performed in MRT models with *SMARCB1* re-expression identified EZH2 and BRD9 as promising therapeutic targets (Erkek et al., 2019; Wang et al., 2019). Moreover, aberrant epigenetic regulation is often causal of the malignant embryonic state of pediatric cancer cells (Lawlor and Thiele, 2012), potentially explaining the sensitivity of different embryonal tumors to drugs targeting epigenetic modifiers (Table 1). Treatment of *in vitro* pediatric tumor models with differentiation agents can recapitulate the effects achieved by driver reversal. However, a durable effect of differentiation therapy can only be acquired through induction of an irreversible growth arrest. As *in vivo* studies have shown, single agent treatment may not suffice to induce terminal differentiation and that combination therapy is required to do so (Hahn et al., 2008; Botrugno et al., 2009; Westerlund et al., 2017; Chen et al., 2018). A powerful tool to identify new (combinations of) drugs are high-throughput drug screens performed on *in vitro* tumor models. Organoids directly derived from patient tumor tissue could provide such models, as they have been shown to closely resemble its parental tissue (Drost and Clevers, 2018). Confirming their potential, an increasing number of reports described that tumor organoids are predictive for patient drug response (Tiriac et al., 2018; Vlachogiannis et al., 2018; Ganesh et al., 2019; Ooft et al., 2019; Yao et al., 2020). Recently, the organoid technology was also successfully applied to several pediatric cancers, including embryonal tumors such as MRT and Wilms tumors (Schutgens et al., 2019; Calandrini et al., 2020). The efficient establishment and cryopreservation of tumor organoid models from primary patient tissue allows for the generation of large patient cohorts stored in organoid biobanks. This is seemingly of particular interest for rare tumors, such as embryonal tumors, for which research material is scarce. In conclusion, the generation of novel and more representative *in vitro* embryonal tumor models is key for the improvement of differentiation therapeutics.

**TABLE 1:** *In vitro* embryonic tumor initiation models and differentiation therapies.

Tumor	Origin	*In vitro* models	Differentiation therapy
Neuroblastoma	Neural crest cells (NCCs)	MYCN overexpression in mouse primary NCCs (Olsen et al., 2017) MYCN/ALK-F1174L overexpression in a mouse NC cell-line (Schulte et al., 2013) Mouse-human chimeras with MYCN overexpression in iPSC-derived hNCCs (Cohen et al., 2020) Engineering human 1p36 deletions in mouse NCCs (García-López et al., 2020)	Retinoic acid treatment (Lone et al., 2016; Westerlund et al., 2017) HDAC inhibitors (Hahn et al., 2008; Frumm et al., 2013) EZH2 inhibitors (Chen et al., 2018)
MRT	Neural crest cells (NCCs)	SMARCB1 knockout in iPSCs (Terada et al., 2019) SMARCB1 knockdown in ESCs (Langer et al., 2019) SMARCB1 knockout in cerebellar organoids (Parisian et al., 2020)	HDAC inhibitors (Muscat et al., 2016) EZH2 inhibitors (Knutson et al., 2013)
Medulloblastoma	Neural progenitor cells	c-MYC overexpression in cerebellar organoids (Ballabio et al., 2020) MYCN overexpression in neuroepithelial stem cells (Huang et al., 2019)	Retinoic acid treatment (Patties et al., 2016) EZH2 inhibitors (Cheng et al., 2020) SHH inhibitors (Ocasio et al., 2019) BET-bromodomain inhibitors (Bandopadhayay et al., 2019)
DIPG	Oligodendrocyte precursor cells	H3K27M mutations in hESC derived NPCs (Funato et al., 2014) ACVR1 mutations in neurospheres (Hoeman et al., 2019)	HDAC inhibitors (Anastas et al., 2019) BET-bromodomain inhibitors (Mohammad et al., 2017)
Retinoblastoma	Cone precursor cells	RB1 depletion in fetal retinal cell cultures (Xu et al., 2014) RB1 depletion in hESC derived retinal organoids (Liu H. et al., 2020; Zheng et al., 2020)	

## Discussion

**FIGURE 1:**
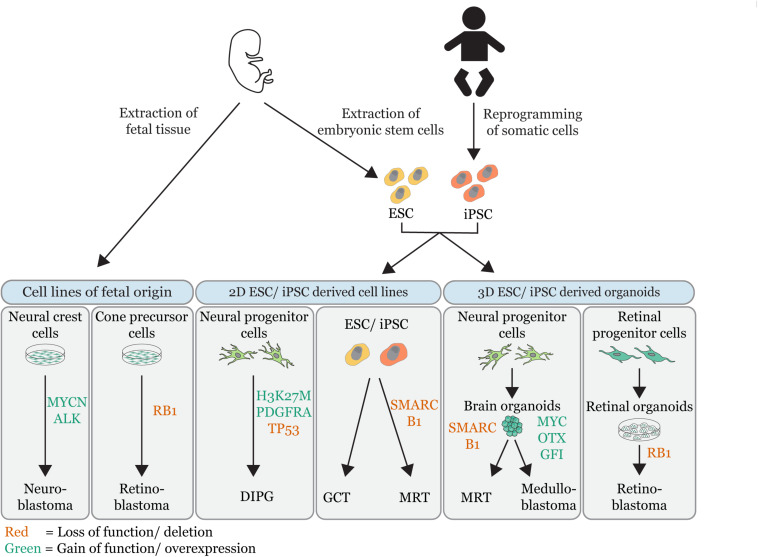
Overview of embryonal tumor modeling techniques. Illustration that summarizes the different *in vitro* approaches used to model embryonal tumors (MRT = malignant rhabdoid tumor; DIPG = diffuse intrinsic pontine glioma, GCT = germ cell tumor). *In vitro* tumor models are grouped by their source: cell lines of fetal origin, 2D embryonic stem cell (ESC) derived or induced pluripotent stem cell derived (iPSC) cell lines and 3D ESC/iPSC derived organoids. The gene-editing of tumor driver events is indicated (red = loss of function/deletions; green = gain of function/overexpression). Permission to reuse and Copyright: Medical illustrations used in in this figure were modified from Servier Medical Art, licensed under a Creative Commons Attribution 3.0 Generic License.

In this review, we have attempted to outline the rapidly developing field of *in vitro* embryonal tumor models and discussed their added value to embryonal tumor research (Figure 1 and Table 1). Still, each model has its intrinsic limitations. For instance, fetal cells can be extracted and cultured from fetal tissues (Xu et al., 2014), but in many cases they do not represent the continuously changing cellular identities found during embryonic development. Alternatively, iPSCs or ESCs cell lines can be deployed to recapitulate these dynamics. Still, even though the spectrum of differentiation protocols is rapidly expanding, many embryonic cell types found *in vivo* cannot yet be captured *in vitro*. Additionally, *in vitro* cultures of ESCs or iPSCs have been shown to be susceptible to “spontaneous” malignant transformation, which can complicate the interpretation of modeling experiments (Ben-David and Benvenisty, 2011). Furthermore, 2D cultures do not capture 3D tissue architecture (Pampaloni et al., 2007). These limitations have been to some extent improved in 3D organoid cultures, which better capture the cell-cell interactions found during embryonic organogenesis (Clevers, 2016). The development of mouse-human chimeras has highlighted the role of the microenvironment in tumor progression (Cohen et al., 2020) and reveals a promising opportunity to bridge the gap of *in vitro* and *in vivo* tumor modeling, as mouse-human chimeras have the advantage of having human cells combined with an *in vivo* murine microenvironment. A good representation of patient tumor evolution remains challenging in *in vitro* models. In patients, tumors originate from a single tumor-initiating cell, wherein a genetic driver event induces aberrant signaling pathways that provide a cell with competitive advantages. Continuous selection of such cells (clonal selection) is thought to form the basis of tumor initiation, progression, and heterogeneity (Navin et al., 2010, 2011). *In vitro* models typically do not reflect the environmental conditions causing clonal selection, as culture conditions are only a simplified version of *in vivo* signaling complexity. Embryonal tumors maintain a fetal identity, which is no longer present in matured tissues (Orbach et al., 2013; Marshall et al., 2014). The characterization of developmental programs in embryonal tumors can therefore give crucial insights into the processes underpinning malignant growth. Single cell transcriptome profiling of tumors and developing tissues has proven to be a promising tool to reveal such processes, which could potentially serve as therapeutic targets (Filbin et al., 2018; Zhang et al., 2019). Similar methods can also be applied to *in vitro* models recapitulating embryonal tumorigenesis, as demonstrated for the retinoblastoma organoid model generated by Liu H. et al. (2020), which has the advantage that it allows for a direct comparison of normal and tumor development.

Although many *in vitro* embryonic cell-derived tumor models have been established over the years, the spectrum is biased toward ectoderm-derived tumors. It seems a matter of time before mesoderm- or endoderm-derived *in vitro* tumor models (e.g., Wilms tumor and hepatoblastoma) will be developed, as the number of culture systems for fetal tissues is rapidly expanding (Low et al., 2019; Ooms et al., 2020; Hendriks et al., 2021).

We are only just beginning to understand the complexity of embryonal tumor development. Although capturing this complexity in a single *in vitro* model might not be feasible, further development of representative *in vitro* cell models recapitulating at least part of it is crucial to gain further insight into the fundamental processes underpinning malignant growth and the development of new therapeutic strategies.

## Author Contributions

LC, IP, and JD wrote the manuscript. JD supervised the work. All authors approved the manuscript for publication.

## Conflict of Interest

The authors declare that the research was conducted in the absence of any commercial or financial relationships that could be construed as a potential conflict of interest.

## Abbreviations

ASC: adult stem cell
DIPG: diffuse intrinsic pontine glioma
ESC: embryonic stem cell
GCT: germ cell tumor
GEMM: genetically engineered mouse model
H3K27M: histone 3 methionine for lysine 27 substitution
iPSC: induced pluripotent stem cell
MRT: malignant rhabdoid tumor
NPC: neural progenitor cell
NCC: neural crest cell
RPC: retinal precursor cell
SWI/SNF: SWItch/Sucrose Non-Fermentable.

## References

[B1] Anastas J. N. Zee B. M. Kalin J. H. Kim M. Guo R. Alexandrescu S. (2019). Re-programing chromatin with a bifunctional LSD1/HDAC inhibitor induces therapeutic differentiation in DIPG. *Cancer Cell* 36 528–544.e10. 10.1016/j.ccell.2019.09.005 31631026

[B2] Ballabio C. Anderle M. Gianesello M. Lago C. Miele E. Cardano M. (2020). Modeling medulloblastoma in vivo and with human cerebellar organoids. *Nat. Commun.* 11:583. 10.1038/s41467-019-13989-3 31996670 PMC6989674

[B3] Bandopadhayay P. Piccioni F. O’Rourke R. Ho P. Gonzalez E. M. Buchan G. (2019). Neuronal differentiation and cell-cycle programs mediate response to BET-bromodomain inhibition in MYC-driven medulloblastoma. *Nat. Commun.* 10:2400. 10.1038/s41467-019-10307-9 31160565 PMC6546744

[B4] Ben-David U. Benvenisty N. (2011). The tumorigenicity of human embryonic and induced pluripotent stem cells. *Nat. Rev. Cancer* 11 268–277. 10.1038/nrc3034 21390058

[B5] Berry R. L. Ozdemir D. D. Aronow B. Lindström N. O. Dudnakova T. Thornburn A. (2015). Deducing the stage of origin of Wilms’ tumours from a developmental series of Wt1-mutant mice. *DMM Dis. Models Mechan.* 8 903–917. 10.1242/dmm.018523 26035382 PMC4527280

[B6] Betters E. Liu Y. Kjaeldgaard A. Sundström E. García-Castro M. I. (2010). Analysis of early human neural crest development. *Dev. Biol.* 344 578–592. 10.1016/j.ydbio.2010.05.012 20478300 PMC2927129

[B7] Betz B. L. Strobeck M. W. Reisman D. N. Knudsen E. S. Weissman B. E. (2002). Re-expression of hSNF5/INI1/BAF47 in pediatric tumor cells leads to G1 arrest associated with induction of p16ink4a and activation of RB. *Oncogene* 21 5193–5203. 10.1038/sj.onc.1205706 12149641

[B8] Bian S. Repic M. Guo Z. Kavirayani A. Burkard T. Bagley J. A. (2018). Genetically engineered cerebral organoids model brain tumor formation. *Nat. Methods* 15 631–639. 10.1038/s41592-018-0070-7 30038414 PMC6071863

[B9] Blakeley P. Fogarty N. M. E. Del Valle I. Wamaitha S. E. Hu T. X. Elder K. (2015). Defining the three cell lineages of the human blastocyst by single-cell RNA-seq. *Development (Cambridge)* 142 3151–3165. 10.1242/dev.123547 26293300 PMC4582176

[B10] Boeva V. Louis-Brennetot C. Peltier A. Durand S. Pierre-Eugène C. Raynal V. (2017). Heterogeneity of neuroblastoma cell identity defined by transcriptional circuitries. *Nat. Genet.* 49 1408–1413. 10.1038/ng.3921 28740262

[B11] Botrugno O. A. Santoro F. Minucci S. (2009). Histone deacetylase inhibitors as a new weapon in the arsenal of differentiation therapies of cancer. *Cancer Lett.* 280 134–144. 10.1016/j.canlet.2009.02.027 19345000

[B12] Calandrini C. Schutgens F. Oka R. Margaritis T. Candelli T. Mathijsen L. (2020). An organoid biobank for childhood kidney cancers that captures disease and tissue heterogeneity. *Nat. Commun.* 11:1310. 10.1038/s41467-020-15155-6 32161258 PMC7066173

[B13] Cavalli F. M. G. Remke M. Rampasek L. Peacock J. Shih D. J. H. Luu B. (2017). Intertumoral heterogeneity within medulloblastoma subgroups. *Cancer Cell* 31 737–754.e6. 10.1016/j.ccell.2017.05.005 28609654 PMC6163053

[B14] Chen L. Alexe G. Dharia N. V. Ross L. Iniguez A. B. Conway A. S. (2018). CRISPR-Cas9 screen reveals a MYCN-amplified neuroblastoma dependency on EZH2. *J. Clin. Invest.* 128 446–462. 10.1172/JCI90793 29202477 PMC5749506

[B15] Chen X. Pappo A. Dyer M. A. (2015). Pediatric solid tumor genomics and developmental pliancy. *Oncogene* 34 5207–5215. 10.1038/onc.2014.474 25639868 PMC4522402

[B16] Cheng Y. Liao S. Xu G. Hu J. Guo D. Du F. (2020). NeuroD1 dictates tumor cell differentiation in medulloblastoma. *Cell Rep.* 31:107782. 10.1016/j.celrep.2020.107782 32579914 PMC7357167

[B17] Clevers H. (2016). Modeling development and disease with organoids. *Cell* 165 1586–1597. 10.1016/j.cell.2016.05.082 27315476

[B18] Cohen M. A. Zhang S. Sengupta S. Ma H. Bell G. W. Horton B. (2020). Formation of human neuroblastoma in mouse-human neural crest chimeras. *Cell Stem Cell* 26 579–592.e6. 10.1016/j.stem.2020.02.001 32142683 PMC7663823

[B19] Cunningham R. M. Walton M. A. Carter P. M. (2018). The major causes of death in children and adolescents in the United States. *N. Eng. J. Med.* 379 2468–2475. 10.1056/nejmsr1804754 30575483 PMC6637963

[B20] Dannenberg J. H. Schuijff L. Dekker M. Van Der Valk M. Te Riele H. (2004). Tissue-specific tumor suppressor activity of retinoblastoma gene homologs p107 and p130. *Genes Dev.* 18 2952–2962. 10.1101/gad.322004 15574596 PMC534655

[B21] De Jong J. Looijenga L. H. J. (2006). Stem cell marker OCT3/4 in tumor biology and germ cell tumor diagnostics: history and future. *Crit. Rev. Oncogen.* 12 171–203. 10.1615/CritRevOncog.v12.i3-4.10 17425502

[B22] Dimaras H. Corson T. W. Cobrinik D. White A. Zhao J. Munier F. L. (2015). Retinoblastoma. *Nat. Rev. Dis. Primers* 1:1502. 10.1038/nrdp.2015.21 27189421 PMC5744255

[B23] Drost J. Clevers H. (2018). Organoids in cancer research. *Nat. Rev. Cancer* 18 407–418. 10.1038/s41568-018-0007-6 29692415

[B24] Dunbar C. E. High K. A. Joung J. K. Kohn D. B. Ozawa K. Sadelain M. (2018). Gene therapy comes of age. *Science* 359:eaan4672. 10.1126/science.aan4672 29326244

[B25] Erkek S. Johann P. D. Finetti M. A. Drosos Y. Chou H. C. Zapatka M. (2019). Comprehensive analysis of chromatin states in atypical teratoid/rhabdoid tumor identifies diverging roles for SWI/SNF and polycomb in gene regulation. *Cancer Cell* 35 95–110.e8. 10.1016/j.ccell.2018.11.014 30595504 PMC6341227

[B26] Filbin M. G. Tirosh I. Hovestadt V. Shaw M. L. Escalante L. E. Mathewson N. D. (2018). Developmental and oncogenic programs in H3K27M gliomas dissected by single-cell RNA-seq. *Science* 360 331–335. 10.1126/science.aao4750 29674595 PMC5949869

[B27] Frumm S. M. Fan Z. P. Ross K. N. Duvall J. R. Gupta S. Verplank L. (2013). Selective HDAC1/HDAC2 inhibitors induce neuroblastoma differentiation. *Chem. Biol.* 20 713–725. 10.1016/j.chembiol.2013.03.020 23706636 PMC3919449

[B28] Funato K. Major T. Lewis P. W. Allis C. D. Tabar V. (2014). Use of human embryonic stem cells to model pediatric gliomas with H3.3K27M histone mutation. *Science* 346 1529–1533. 10.1126/science.1253799 25525250 PMC4995593

[B29] Ganesh K. Wu C. O’Rourke K. P. Szeglin B. C. Zheng Y. Sauvé C. E. G. (2019). A rectal cancer organoid platform to study individual responses to chemoradiation. *Nat. Med.* 25 1607–1614. 10.1038/s41591-019-0584-2 31591597 PMC7385919

[B30] García-López J. Wallace K. Otero J. H. Olsen R. Wang Y. dong (2020). Large 1p36 deletions affecting arid1a locus facilitate mycn-driven oncogenesis in neuroblastoma. *Cell Rep.* 30 454–464.e5. 10.1016/j.celrep.2019.12.048 31940489 PMC9022217

[B31] Gibson P. Tong Y. Robinson G. Thompson M. C. Currle D. S. Eden C. (2010). Subtypes of medulloblastoma have distinct developmental origins. *Nature* 468 1095–1099. 10.1038/nature09587 21150899 PMC3059767

[B32] Grammel D. Warmuth-Metz M. Von Bueren A. O. Kool M. Pietsch T. Kretzschmar H. A. (2012). Sonic hedgehog-associated medullobla stoma arising from the cochlear nuclei of the brainstem. *Acta Neuropathol.* 123 601–614. 10.1007/s00401-012-0961-0 22349907

[B33] Gröbner S. N. Worst B. C. Weischenfeldt J. Buchhalter I. Kleinheinz K. Rudneva V. A. (2018). The landscape of genomic alterations across childhood cancers. *Nature* 555 321–327. 10.1038/nature25480 29489754

[B34] Hahn C. K. Ross K. N. Warrington I. M. Mazitschek R. Kanegai C. M. Wright R. D. (2008). Expression-based screening identifies the combination of histone deacetylase inhibitors and retinoids for neuroblastoma differentiation. *Proc. Natl. Acad. Sci. U S A.* 105 9751–9756. 10.1073/pnas.0710413105 18607002 PMC2474517

[B35] Han Z. Y. Richer W. Fréneaux P. Chauvin C. Lucchesi C. Guillemot D. (2016). The occurrence of intracranial rhabdoid tumours in mice depends on temporal control of Smarcb1 inactivation. *Nat. Commun.* 7:10421. 10.1038/ncomms10421 26818002 PMC4738337

[B36] Hasselblatt M. Nagel I. Oyen F. Bartelheim K. Russell R. B. Schüller U. (2014). SMARCA4-mutated atypical teratoid/rhabdoid tumors are associated with inherited germline alterations and poor prognosis. *Acta Neuropathol.* 128 453–456. 10.1007/s00401-014-1323-x 25060813

[B37] Hendriks D. Artegiani B. Hu H. Chuva, de Sousa Lopes S. Clevers H. (2021). Establishment of human fetal hepatocyte organoids and CRISPR–Cas9-based gene knockin and knockout in organoid cultures from human liver. *Nat. Protocols* 16 182–217. 10.1038/s41596-020-00411-2 33247284

[B38] Henriksen J. R. Haug B. H. Buechner J. Tømte E. Løkke C. Flaegstad T. (2011). Conditional expression of retrovirally delivered anti-MYCN shRNA as an in vitro model system to study neuronal differentiation in MYCN-amplified neuroblastoma. *BMC Dev. Biol.* 11:1. 10.1186/1471-213X-11-1 21194500 PMC3022612

[B39] Hoeman C. M. Cordero F. J. Hu G. Misuraca K. Romero M. M. Cardona H. J. (2019). ACVR1 R206H cooperates with H3.1K27M in promoting diffuse intrinsic pontine glioma pathogenesis. *Nat. Commun.* 10:1023. 10.1038/s41467-019-08823-9 30833574 PMC6399349

[B40] Hossain M. M. Banik N. L. Ray S. K. (2013). N-Myc knockdown and apigenin treatment controlled growth of malignant neuroblastoma cells having N-Myc amplification. *Gene* 529 27–36. 10.1016/j.gene.2013.07.094 23941992 PMC3805111

[B41] Hovestadt V. Smith K. S. Bihannic L. Filbin M. G. Shaw M. K. L. Baumgartner A. (2019). Resolving medulloblastoma cellular architecture by single-cell genomics. *Nature* 572 74–79. 10.1038/s41586-019-1434-6 31341285 PMC6754173

[B42] Hu Q. Gao F. Tian W. Ruteshouser E. C. Wang Y. Lazar A. (2011). Wt1 ablation and Igf2 upregulation in mice result in wilms tumors with elevated ERK1/2 phosphorylation. *J. Clin. Invest.* 121 174–183. 10.1172/JCI43772 21123950 PMC3007149

[B43] Huang L. Mokkapati S. Hu Q. Ruteshouser E. C. Hicks M. J. Huff V. (2016). Nephron Progenitor but not stromal progenitor cells give rise to wilms tumors in mouse models with β-Catenin activation or Wt1 ablation and Igf2 upregulation. *Neoplasia (United States)* 18 71–81. 10.1016/j.neo.2015.12.001 26936393 PMC5005262

[B44] Huang M. Tailor J. Zhen Q. Gillmor A. H. Miller M. L. Weishaupt H. (2019). Engineering genetic predisposition in human neuroepithelial stem cells recapitulates medulloblastoma tumorigenesis. *Cell Stem Cell* 25 433–446.e7. 10.1016/j.stem.2019.05.013 31204176 PMC6731167

[B45] Jessa S. Blanchet-Cohen A. Krug B. Vladoiu M. Coutelier M. Faury D. (2019). Stalled developmental programs at the root of pediatric brain tumors. *Nat. Genet.* 51 1702–1713. 10.1038/s41588-019-0531-7 31768071 PMC6885128

[B46] Jiang R. Xue S. Jin Z. (2011). Stable knockdown of MYCN by lentivirus-based RNAi inhibits human neuroblastoma cells growth in vitro and in vivo. *Biochem. Biophys. Res. Commun.* 410 364–370. 10.1016/j.bbrc.2011.06.020 21683062

[B47] Johnsen J. I. Dyberg C. Wickström M. (2019). Neuroblastoma—a neural crest derived embryonal malignancy. *Front. Mol. Neurosci.* 12:9. 10.3389/fnmol.2019.00009 30760980 PMC6361784

[B48] Kang J. H. Rychahou P. G. Ishola T. A. Qiao J. Evers B. M. Chung D. H. (2006). MYCN silencing induces differentiation and apoptosis in human neuroblastoma cells. *Biochem. Biophys. Res. Commun.* 351 192–197. 10.1016/j.bbrc.2006.10.020 17055458 PMC2708968

[B49] Kattner P. Strobel H. Khoshnevis N. Grunert M. Bartholomae S. Pruss M. (2019). Compare and contrast: pediatric cancer versus adult malignancies. *Cancer Metastasis Rev.* 38 673–682. 10.1007/s10555-019-09836-y 31832830

[B50] Khuong-Quang D. A. Buczkowicz P. Rakopoulos P. Liu X. Y. Fontebasso A. M. Bouffet E. (2012). K27M mutation in histone H3.3 defines clinically and biologically distinct subgroups of pediatric diffuse intrinsic pontine gliomas. *Acta Neuropathol.* 124 439–447. 10.1007/s00401-012-0998-0 22661320 PMC3422615

[B51] Knutson S. K. Warholic N. M. Wigle T. J. Klaus C. R. Allain C. J. Raimondi A. (2013). Durable tumor regression in genetically altered malignant rhabdoid tumors by inhibition of methyltransferase EZH2. *Proc. Natl. Acad. Sci. U S A.* 110 7922–7927. 10.1073/pnas.1303800110 23620515 PMC3651445

[B52] Kreidberg J. A. Sariola H. Loring J. M. Maeda M. Pelletier J. Housman D. (1993). WT-1 is required for early kidney development. *Cell* 74 679–691. 10.1016/0092-8674(93)90515-R 8395349

[B53] Kuwahara A. Ozone C. Nakano T. Saito K. Eiraku M. Sasai Y. (2015). Generation of a ciliary margin-like stem cell niche from self-organizing human retinal tissue. *Nat. Commun.* 6:6286. 10.1038/ncomms7286 25695148

[B54] Langer L. F. Ward J. M. Archer T. K. (2019). Tumor suppressor SMARCB1 suppresses super-enhancers to govern hESC lineage determination. *eLife* 8:e45672. 10.7554/eLife.45672 31033435 PMC6538374

[B55] Lawlor E. R. Thiele C. J. (2012). Epigenetic changes in pediatric solid tumors: promising new targets. *Clin. Cancer Res.* 18 2768–2779. 10.1158/1078-0432.CCR-11-1921 22589485 PMC3691809

[B56] Lee A. S. Tang C. Rao M. S. Weissman I. L. Wu J. C. (2013). Tumorigenicity as a clinical hurdle for pluripotent stem cell therapies. *Nat. Med.* 19 998–1004. 10.1038/nm.3267 23921754 PMC3967018

[B57] Lee E. Y. H. P. Chang C. Y. Hu N. Wang Y. C. J. Lai C. C. Herrup K. (1992). Mice deficient for Rb are nonviable and show defects in neurogenesis and haematopoiesis. *Nature* 359 288–294. 10.1038/359288a0 1406932

[B58] Lee R. S. Stewart C. Carter S. L. Ambrogio L. Cibulskis K. Sougnez C. (2012). A remarkably simple genome underlies highly malignant pediatric rhabdoid cancers. *J. Clin. Invest.* 122 2983–2988. 10.1172/JCI64400 22797305 PMC3408754

[B59] Liu G. David B. T. Trawczynski M. Fessler R. G. (2020). Advances in pluripotent stem cells: history, mechanisms, technologies, and applications. *Stem Cell Rev. Rep.* 16 3–32. 10.1007/s12015-019-09935-x 31760627 PMC6987053

[B60] Liu H. Sun Q. Sun Y. Zhang J. Yuan H. Pang S. (2017). MELK and EZH2 cooperate to regulate medulloblastoma cancer stem-like cell proliferation and differentiation. *Mol. Cancer Res.* 15 1275–1286. 10.1158/1541-7786.MCR-17-0105 28536141

[B61] Liu H. Zhang Y. Zhang Y.-Y. Li Y.-P. Hua Z.-Q. Zhang C.-J. (2020). Human embryonic stem cell-derived organoid retinoblastoma reveals a cancerous origin. *Proc. Natl. Acad. Sci.* 117 33628–33638. 10.1073/pnas.2011780117 33318192 PMC7776986

[B62] Lone A. M. Dar N. J. Hamid A. Shah W. A. Ahmad M. Bhat B. A. (2016). Promise of retinoic acid-triazolyl derivatives in promoting differentiation of neuroblastoma cells. *ACS Chem. Neurosci.* 7 82–89. 10.1021/acschemneuro.5b00267 26551203

[B63] Low J. H. Li P. Chew E. G. Y. Zhou B. Suzuki K. Zhang T. (2019). Generation of human PSC-Derived kidney organoids with patterned nephron segments and a de novo vascular network. *Cell Stem Cell* 25 373–387.e9. 10.1016/j.stem.2019.06.009 31303547 PMC6731150

[B64] Luo C. Lancaster M. A. Castanon R. Nery J. R. Knoblich J. A. Ecker J. R. (2016). Cerebral organoids recapitulate epigenomic signatures of the human fetal brain. *Cell Rep.* 17 3369–3384. 10.1016/j.celrep.2016.12.001 28009303 PMC5495578

[B65] MacPherson D. Sage J. Kim T. Ho D. McLaughlin M. E. Jacks T. (2004). Cell type-specific effects of Rb deletion in the murine retina. *Genes Dev.* 18 1681–1694. 10.1101/gad.1203304 15231717 PMC478190

[B66] Marshall G. M. Carter D. R. Cheung B. B. Liu T. Mateos M. K. Meyerowitz J. G. (2014). The prenatal origins of cancer. *Nat. Rev. Cancer* 14 277–289. 10.1038/nrc3679 24599217 PMC4041218

[B67] Maurer J. Fuchs S. Jäger R. Kurz B. Sommer L. Schorle H. (2007). Establishment and controlled differentiation of neural crest stem cell lines using conditional transgenesis. *Differentiation* 75 580–591. 10.1111/j.1432-0436.2007.00164.x 17381545

[B68] Mohammad F. Weissmann S. Leblanc B. Pandey D. P. Højfeldt J. W. Comet I. (2017). EZH2 is a potential therapeutic target for H3K27M-mutant pediatric gliomas. *Nat. Med.* 23 483–492. 10.1038/nm.4293 28263309

[B69] Montavon G. Jauquier N. Coulon A. Peuchmaur M. Flahaut M. Bourloud K. B. (2014). Wild-type ALK and activating ALK-R1275Q and ALK-F1174L mutations upregulate Myc and initiate tumor formation in murine neural crest progenitor cells. *Oncotarget* 5 4452–4466. 10.18632/oncotarget.2036 24947326 PMC4147337

[B70] Muguruma K. Nishiyama A. Kawakami H. Hashimoto K. Sasai Y. (2015). Self-organization of polarized cerebellar tissue in 3D culture of human pluripotent stem cells. *Cell Rep.* 10 537–550. 10.1016/j.celrep.2014.12.051 25640179

[B71] Muscat A. Popovski D. Jayasekara W. S. N. Rossello F. J. Ferguson M. Marini K. D. (2016). Low-dose histone deacetylase inhibitor treatment leads to tumor growth arrest and multi-lineage differentiation of malignant rhabdoid tumors. *Clin. Cancer Res.* 22 3560–3570. 10.1158/1078-0432.CCR-15-2260 26920892

[B72] Nakayama R. T. Pulice J. L. Valencia A. M. McBride M. J. McKenzie Z. M. Gillespie M. A. (2017). SMARCB1 is required for widespread BAF complex-mediated activation of enhancers and bivalent promoters. *Nat. Genet.* 49 1613–1623. 10.1038/ng.3958 28945250 PMC5803080

[B73] Navin N. Kendall J. Troge J. Andrews P. Rodgers L. McIndoo J. (2011). Tumour evolution inferred by single-cell sequencing. *Nature* 472 90–95. 10.1038/nature09807 21399628 PMC4504184

[B74] Navin N. Krasnitz A. Rodgers L. Cook K. Meth J. Kendall J. (2010). Inferring tumor progression from genomic heterogeneity. *Genome Res.* 20 68–80. 10.1101/gr.099622.109 19903760 PMC2798832

[B75] Ocasio J. Babcock B. Malawsky D. Weir S. J. Loo L. Simon J. M. (2019). scRNA-seq in medulloblastoma shows cellular heterogeneity and lineage expansion support resistance to SHH inhibitor therapy. *Nat. Commun.* 10:5829. 10.1038/s41467-019-13657-6 31863004 PMC6925218

[B76] Olsen R. R. Otero J. H. García-López J. Wallace K. Finkelstein D. Rehg J. E. (2017). MYCN induces neuroblastoma in primary neural crest cells. *Oncogene* 36 5075–5082. 10.1038/onc.2017.128 28459463 PMC5582212

[B77] Ooft S. N. Weeber F. Dijkstra K. K. McLean C. M. Kaing S. van Werkhoven E. (2019). Patient-derived organoids can predict response to chemotherapy in metastatic colorectal cancer patients. *Sci. Trans. Med.* 11:eaay2574. 10.1126/scitranslmed.aay2574 31597751

[B78] Ooms A. H. A. G. Calandrini C. de Krijger R. R. Drost J. (2020). Organoid models of childhood kidney tumours. *Nat. Rev. Urol.* 17 311–313. 10.1038/s41585-020-0315-y 32242130

[B79] Oosterhuis J. W. Looijenga L. H. J. (2019). Human germ cell tumours from a developmental perspective. *Nat. Rev. Cancer* 19 522–537. 10.1038/s41568-019-0178-9 31413324

[B80] O’Rahilly R. Müller F. (2007). The development of the neural crest in the human. *J. Anatomy* 211 335–351. 10.1111/j.1469-7580.2007.00773.x 17848161 PMC2375817

[B81] Orbach D. Sarnacki S. Brisse H. J. Gauthier-Villars M. Jarreau P. H. Tsatsaris V. (2013). Neonatal cancer. *Lancet Oncol.* 14 e609–e620. 10.1016/S1470-2045(13)70236-5 24275134

[B82] Pampaloni F. Reynaud E. G. Stelzer E. H. K. (2007). The third dimension bridges the gap between cell culture and live tissue. *Nat. Rev. Mol. Cell Biol.* 8 839–845. 10.1038/nrm2236 17684528

[B83] Parisian A. D. Koga T. Miki S. Johann P. D. Kool M. Crawford J. R. (2020). SMARCB1 loss interacts with neuronal differentiation state to block maturation and impact cell stability. *Genes Dev.* 34 1316–1329. 10.1101/gad.339978.120 32912900 PMC7528703

[B84] Patties I. Kortmann R. D. Menzel F. Glasow A. (2016). Enhanced inhibition of clonogenic survival of human medulloblastoma cells by multimodal treatment with ionizing irradiation, epigenetic modifiers, and differentiation-inducing drugs. *J. Exp. Clin. Cancer Res.* 35:1. 10.1186/s13046-016-0376-1 27317342 PMC4912728

[B85] Puisieux A. Pommier R. M. Morel A. P. Lavial F. (2018). Cellular pliancy and the multistep process of tumorigenesis. *Cancer Cell* 33 164–172. 10.1016/j.ccell.2018.01.007 29438693

[B86] Rahal Z. Abdulhai F. Kadara H. Saab R. (2018). Genomics of adult and pediatric solid tumors. *Am. J. Cancer Res.* 8 1356–1386. 30210910 PMC6129500

[B87] Reiff T. Tsarovina K. Majdazari A. Schmidt M. Del Pino I. Rohrer H. (2010). Neuroblastoma Phox2b variants stimulate proliferation and dedifferentiation of immature sympathetic neurons. *J. Neurosci.* 30 905–915. 10.1523/JNEUROSCI.5368-09.2010 20089899 PMC6633096

[B88] Robanus-Maandag E. Dekker M. Van Der Valk M. Carrozza M. L. Jeanny J. C. Dannenberg J. H. (1998). p107 is a suppressor of retinoblastoma development in pRB-deficient mice. *Genes Dev.* 12 1599–1609. 10.1101/gad.12.11.1599 9620848 PMC316874

[B89] Schulte J. H. Lindner S. Bohrer A. Maurer J. De Preter K. Lefever S. (2013). MYCN and ALKF1174L are sufficient to drive neuroblastoma development from neural crest progenitor cells. *Oncogene* 32 1059–1065. 10.1038/onc.2012.106 22484425

[B90] Schutgens F. Rookmaaker M. B. Margaritis T. Rios A. Ammerlaan C. Jansen J. (2019). Tubuloids derived from human adult kidney and urine for personalized disease modeling. *Nat. Biotechnol.* 37 303–313. 10.1038/s41587-019-0048-8 30833775

[B91] Schwartzentruber J. Korshunov A. Liu X. Y. Jones D. T. W. Pfaff E. Jacob K. (2012). Driver mutations in histone H3.3 and chromatin remodelling genes in paediatric glioblastoma. *Nature* 482 226–231. 10.1038/nature10833 22286061

[B92] Shain A. H. Pollack J. R. (2013). The spectrum of SWI/SNF mutations, ubiquitous in human cancers. *PLoS One* 8:e55119. 10.1371/journal.pone.0055119 23355908 PMC3552954

[B93] Siegel R. L. Miller K. D. Jemal A. (2016). Cancer statistics, 2016. *CA: Cancer J. Clin.* 66 7–30. 10.3322/caac.21332 26742998

[B94] Stemple D. L. Anderson D. J. (1992). Isolation of a stem cell for neurons and glia from the mammalian neural crest. *Cell* 71 973–985. 10.1016/0092-8674(92)90393-Q 1458542

[B95] Stratton M. R. Campbell P. J. Futreal P. A. (2009). The cancer genome. *Nature* 458 719–724. 10.1038/nature07943 19360079 PMC2821689

[B96] Sun Y. Xu C. Pan C. Chen X. Geng Y. Wu Y. (2019). Diffuse intrinsic pontine gliomas exhibit cell biological and molecular signatures of fetal hindbrain-derived neural progenitor cells. *Neurosci. Bull.* 35 216–224. 10.1007/s12264-018-00329-6 30607770 PMC6426892

[B97] Terada Y. Jo N. Arakawa Y. Sakakura M. Yamada Y. Ukai T. (2019). Human pluripotent stem cell-derived tumor model uncovers the embryonic stem cell signature as a key driver in atypical teratoid/rhabdoid tumor. *Cell Rep.* 26 2608–2621.e6. 10.1016/j.celrep.2019.02.009 30840885

[B98] Theunissen T. W. Jaenisch R. (2017). Mechanisms of gene regulation in human embryos and pluripotent stem cells. *Development (Cambridge)* 144 4496–4509. 10.1242/dev.157404 29254992 PMC5769625

[B99] Thomas J. Adegboyega P. Iloabachie K. Mooring J. W. Lian T. (2011). Sinonasal teratocarcinosarcoma with yolk sac elements: a neoplasm of somatic or germ cell origin? *Annals Diagn. Pathol.* 15 135–139. 10.1016/j.anndiagpath.2010.01.004 20952296

[B100] Tiriac H. Belleau P. Engle D. D. Plenker D. Deschênes A. Somerville T. D. D. (2018). Organoid profiling identifies common responders to chemotherapy in pancreatic cancer. *Cancer Discovery* 8 1112–1129. 10.1158/2159-8290.CD-18-0349 29853643 PMC6125219

[B101] Visvader J. E. (2011). Cells of origin in cancer. *Nature* 469 314–322. 10.1038/nature09781 21248838

[B102] Vitte J. Gao F. Coppola G. Judkins A. R. Giovannini M. (2017). Timing of Smarcb1 and Nf2 inactivation determines schwannoma versus rhabdoid tumor development. *Nat. Commun.* 8:300. 10.1038/s41467-017-00346-5 28824165 PMC5563506

[B103] Vlachogiannis G. Hedayat S. Vatsiou A. Jamin Y. Fernández-Mateos J. Khan K. (2018). Patient-derived organoids model treatment response of metastatic gastrointestinal cancers. *Science* 359 920–926. 10.1126/science.aao2774 29472484 PMC6112415

[B104] Vladoiu M. C. El-Hamamy I. Donovan L. K. Farooq H. Holgado B. L. Sundaravadanam Y. (2019). Childhood cerebellar tumours mirror conserved fetal transcriptional programs. *Nature* 572 67–73. 10.1038/s41586-019-1158-7 31043743 PMC6675628

[B105] Vogelstein B. Papadopoulos N. Velculescu V. E. Zhou S. Diaz L. A. Kinzler K. W. (2013). Cancer genome landscapes. *Science* 340 1546–1558. 10.1126/science.1235122 23539594 PMC3749880

[B106] Wang X. Lee R. S. Alver B. H. Haswell J. R. Wang S. Mieczkowski J. (2017). SMARCB1-mediated SWI/SNF complex function is essential for enhancer regulation. *Nat. Genet.* 49 289–295. 10.1038/ng.3746 27941797 PMC5285474

[B107] Wang X. Wang S. Troisi E. C. Howard T. P. Haswell J. R. Wolf B. K. (2019). BRD9 defines a SWI/SNF sub-complex and constitutes a specific vulnerability in malignant rhabdoid tumors. *Nat. Commun.* 10:1881. 10.1038/s41467-019-09891-7 31015438 PMC6479050

[B108] Westerlund I. Shi Y. Toskas K. Fell S. M. Li S. Surova O. (2017). Combined epigenetic and differentiation-based treatment inhibits neuroblastoma tumor growth and links HIF2α to tumor suppression. *Proc. Natl. Acad. Sci. U S A.* 114 E6137–E6146. 10.1073/pnas.1700655114 28696319 PMC5544284

[B109] Westermark U. K. Wilhelm M. Frenzel A. Henriksson M. A. (2011). The MYCN oncogene and differentiation in neuroblastoma. *Sem. Cancer Biol.* 21 256–266. 10.1016/j.semcancer.2011.08.001 21849159

[B110] Wikenheiser-Brokamp K. A. (2006). Retinoblastoma family proteins: insights gained through genetic manipulation of mice. *Cell. Mol. Life Sci.* 63 767–780. 10.1007/s00018-005-5487-3 16465443 PMC11136128

[B111] Wilson B. G. Roberts C. W. M. (2011). SWI/SNF nucleosome remodellers and cancer. *Nat. Rev. Cancer* 11 481–492. 10.1038/nrc3068 21654818

[B112] Wu G. Broniscer A. McEachron T. A. Lu C. Paugh B. S. Becksfort J. (2012). Somatic histone H3 alterations in pediatric diffuse intrinsic pontine gliomas and non-brainstem glioblastomas. *Nat. Genet.* 44 251–253. 10.1038/ng.1102 22286216 PMC3288377

[B113] Xu X. L. Singh H. P. Wang L. Qi D. L. Poulos B. K. Abramson D. H. (2014). Rb suppresses human cone-precursor-derived retinoblastoma tumours. *Nature* 514 385–388. 10.1038/nature13813 25252974 PMC4232224

[B114] Yao Y. Xu X. Yang L. Zhu J. Wan J. Shen L. (2020). Patient-Derived organoids predict chemoradiation responses of locally advanced rectal cancer. *Cell Stem Cell* 26 17–26.e6. 10.1016/j.stem.2019.10.010 31761724

[B115] Young M. D. Mitchell T. J. Custers L. Margaritis T. Morales F. Kwakwa K. (2020). Single cell derived mRNA signals across human kidney tumors. *BioRxiv* [preprint] 10.1101/2020.03.19.998815 PMC8222373 34162837

[B116] Young M. D. Mitchell T. J. Vieira Braga F. A. Tran M. G. B. Stewart B. J. Ferdinand J. R. (2018). Single-cell transcriptomes from human kidneys reveal the cellular identity of renal tumors. *Science* 361 594–599. 10.1126/science.aat1699 30093597 PMC6104812

[B117] Zagozewski J. Shahriary G. M. Morrison L. C. Saulnier O. Stromecki M. Fresnoza A. (2020). An OTX2-PAX3 signaling axis regulates Group 3 medulloblastoma cell fate. *Nat. Commun.* 11:3627. 10.1038/s41467-020-17357-4 32686664 PMC7371715

[B118] Zhang L. He X. Liu X. Zhang F. Huang L. F. Potter A. S. (2019). Single-Cell transcriptomics in medulloblastoma reveals tumor-initiating progenitors and oncogenic cascades during tumorigenesis and relapse. *Cancer Cell* 36 302–318.e7. 10.1016/j.ccell.2019.07.009 31474569 PMC6760242

[B119] Zheng C. Schneider J. W. Hsieh J. (2020). Role of RB1 in human embryonic stem cell-derived retinal organoids. *Dev. Biol.* 462 197–207. 10.1016/j.ydbio.2020.03.011 32197890 PMC7314500

[B120] Zhong X. Gutierrez C. Xue T. Hampton C. Vergara M. N. Cao L. H. (2014). Generation of three-dimensional retinal tissue with functional photoreceptors from human iPSCs. *Nat. Commun.* 5:4047. 10.1038/ncomms5047 24915161 PMC4370190

